# Key Ethical Issues Discussed at CDC-Sponsored International, Regional Meetings to Explore Cultural Perspectives and Contexts on Pandemic Influenza Preparedness and Response

**DOI:** 10.15171/ijhpm.2016.55

**Published:** 2016-05-17

**Authors:** Aun Lor, James C. Thomas, Drue H. Barrett, Leonard W. Ortmann, Dionisio J. Herrera Guibert

**Affiliations:** ^1^Center for Global Health, Centers for Disease Control and Prevention, Atlanta, GA, USA.; ^2^Gilllings School of Global Public Health, University of North Carolina, Chapel Hill, NC, USA.; ^3^Office of Science Integrity, Office of the Associate Director for Science, Centers for Disease Control and Prevention, Atlanta, GA, USA.; ^4^Training Programs in Epidemiology and Public Health Interventions Network, Task Force for Global Health Inc., Atlanta, GA, USA.

**Keywords:** Public Health Ethics, Culture, Influenza, Pandemic Preparedness, Global Health, Emergency Response

## Abstract

**Background:** Recognizing the importance of having a broad exploration of how cultural perspectives may shape thinking about ethical considerations, the Centers for Disease Control and Prevention (CDC) funded four regional meetings in Africa, Asia, Latin America, and the Eastern Mediterranean to explore these perspectives relevant to pandemic influenza preparedness and response. The meetings were attended by 168 health professionals, scientists, academics, ethicists, religious leaders, and other community members representing 40 countries in these regions.

**Methods:** We reviewed the meeting reports, notes and stories and mapped outcomes to the key ethical challenges for pandemic influenza response described in the World Health Organization’s (WHO’s) guidance, Ethical Considerations in Developing a Public Health Response to Pandemic Influenza: transparency and public engagement, allocation of resources, social distancing, obligations to and of healthcare workers, and international collaboration.

**Results:** The important role of transparency and public engagement were widely accepted among participants. However, there was general agreement that no "one size fits all" approach to allocating resources can address the variety of economic, cultural and other contextual factors that must be taken into account. The importance of social distancing as a tool to limit disease transmission was also recognized, but the difficulties associated with this measure were acknowledged. There was agreement that healthcare workers often have competing obligations and that government has a responsibility to assist healthcare workers in doing their job by providing appropriate training and equipment. Finally, there was agreement about the importance of international collaboration for combating global health threats.

**Conclusion:** Although some cultural differences in the values that frame pandemic preparedness and response efforts were observed, participants generally agreed on the key ethical principles discussed in the WHO’s guidance. Most significantly the input gathered from these regional meetings pointed to the important role that procedural ethics can play in bringing people and countries together to respond to the shared health threat posed by a pandemic influenza despite the existence of cultural differences.

## Background

Before the 2014 Ebola outbreak in West Africa captured the world’s attention, one of the most feared yet widely anticipated events in public health was a pandemic of highly pathogenic influenza. In the 20th century, there were three notable influenza pandemics – the “Spanish flu” (H1N1 virus) in 1918 and 1919, which resulted in approximately 50 million deaths worldwide^1^; the “Asian flu” (H2N2 virus) in 1957-1958, which resulted in 1-2 million deaths worldwide^2,3^; and the “Hong Kong flu” (H3N2 virus) in 1968, which resulted in 1 million deaths worldwide.^4,5^ In the late 1990s and in early 2000s, concern focused on the spread of avian influenza virus H5N1 to humans. The first cases of human infection with H5N1 were reported in 1997 in Hong Kong (18 cases of which 6 were fatal).^6-9^ Fears about H5N1 were heightened in 2003 when the virus was found to be responsible for serious disease and death in humans. Nearly 650 human cases of H5N1 have been reported from 15 countries since 2003 through October 2015.^4^

Other outbreaks led to questions about preparedness. The outbreak of severe acute respiratory syndrome (SARS) in 2003 contributed to growing concern about the world’s ability to prepare for and respond to a worldwide epidemic. SARS, first reported in Asia in February 2003, is a viral respiratory illness caused by a coronavirus. The illness spread rapidly to more than two dozen countries in North America, South America, Europe, and Asia before the SARS global outbreak was contained in 2004.^10^ The reemergence of the H1N1 virus during the 2009-2010 influenza season,^11^ the emergence of the Middle East Respiratory Syndrome Coronavirus (MERS-CoV) in 2012,^12^ and the cases of severe illness in humans from a new avian influenza A (H7N9) virus in 2013^13^ heightened concerns about the need to be prepared for pandemics.

An influenza pandemic results in a sudden surge of people with acute health needs, placing extra burden on health resources already overstretched in many places. The severity and suddenness of these burdens can create ethical tensions along a number of fronts. One such tension to which policy-makers already have given considerable attention is the ethical allocation of scarce supplies of antivirals, vaccines, respirators, and personal protective equipment.^14-17^ Healthcare workers will encounter challenging ethical dilemmas involving their professional duties to patients and their strong competing obligation to protect and care for themselves and their family. The employers of these workers will have obligations to minimize risks to their employees, while countries will have obligations relating to international collaboration that can compete with domestic priorities.

At the request of member states, the World Health Organization (WHO) convened an international group in 2006 to identify common ethical concerns in preparing for and responding to a pandemic influenza and to provide preliminary guidance on how to address these issues. This resulted in the 2007 release of *Ethical Considerations in Developing a Public Health Response to Pandemic Influenza*.^18^ In addition to discussing general ethical considerations (eg, balancing rights, interests and values, transparency and community engagement), the WHO ethics guidance discussed issues relating to priority setting and equitable access to therapeutic and prophylactic measures; use of isolation, quarantine, border control and social distancing measures; and the role and obligations of and to healthcare workers. An overriding theme for the WHO guidance was the need for international cooperation and the importance of taking into account the contextual and cultural considerations of particular countries or regions. The WHO document notes that “ethical considerations will be shaped by the local context and cultural values.”^18^

CDC also developed ethical guidance relating to pandemic influenza.^19,20^ This guidance focused on ethical issues relating to allocation of scarce resources and use of public health interventions which may limit individual liberties. In addition to the WHO and CDC documents, there is considerable literature devoted to ethical considerations in pandemic influenza preparedness and response.^21-31^ However, whereas most of this literature assumes a liberal democratic perspective in considering ethics and values, many countries that would partner in a global pandemic response are more hierarchically structured. In hierarchical societies, citizens tend to implicitly expect and trust decisions from their leaders. Notwithstanding that liberal democratic societies emphasize individual autonomy, collective decision-making and the interrelatedness and interdependence of community members are fundamental to every human society.^32^ Because in hierarchic societies these factors have greater visibility, in such societies the success of public health interventions will depend on engaging recognized tribal, community or religious leaders in decision-making.

Recognizing the importance of having a broader exploration of how cultural perspectives may shape thinking about ethical considerations, CDC sponsored meetings in Africa, Asia, Latin America, and the Eastern Mediterranean to explore various cultural perspectives relevant to pandemic influenza preparedness and response. The meeting in Africa was held in collaboration with WHO and the African Field Epidemiology Network (AFENET) in Kampala, Uganda on August 11-15, 2008. The meeting in Asia was held in collaboration with WHO, the Training in Epidemiology and Public Health Interventions Network (TEPHINET), and the South Asia Field Epidemiology and Technology Network (SAFETYNET) in Hanoi, Vietnam on March 22-25, 2010. The meeting in Latin America was held in collaboration with TEPHINET in Santo Domingo, Dominican Republic on July 27-29, 2011. The Eastern Mediterranean meeting was held in collaboration with TEPHINET and the Eastern Mediterranean Public Health Network (EMPHNET 2015) in Sharm El Sheikh, Egypt on December 5-6, 2011. The key objectives for all four meetings were to: (1) identify cultural-specific ethical challenges in pandemic influenza detection and control, (2) explore approaches for addressing these ethical challenges, including how to best integrate ethical considerations into country/regional pandemic influenza preparedness and response guidelines and implementation strategies, and (3) begin establishing a social network to foster continued discussion about ethical issues in the practice of public health.

The meetings were attended by government health officials and policy-makers, public health practitioners, scientists from academic and research institutions, epidemiologists, philosophers, ethicists, religious leaders, and representatives of international aid and health organizations. The African meeting was attended by 71 people, including representatives from 12 African countries (Nigeria, South Sudan, South Africa, Zimbabwe, Togo, Mali, Cameroun, Burkina Faso, Tanzania, Kenya, Egypt, and Uganda). The Asian meeting was attended by 30 people, including representatives from 9 countries (Cambodia, China, India, Indonesia, Laos, Myanmar, Philippines, Thailand, and Vietnam). The Latin America meeting was attended by 33 people, including representatives from 11 Latin American countries (Argentina, Brazil, Columbia, Costa Rica, Dominican Republic, El Salvador, Guatemala, Honduras, Mexico, Panama, and Peru). The Eastern Mediterranean meeting was attended by 34 people, including representatives from 8 countries (Egypt, Afghanistan, Pakistan, Sudan, Yemen, Iraq, Jordan, and Morocco).

## Methods

For this paper, we reviewed the reports, notes, and stories resulted from the four regional meetings. We mapped outcomes from the meetings to five key ethical challenges identified in the WHO guidance: transparency and public engagement, allocation of resources, social distancing, obligations to and of healthcare workers, and international collaboration. We report here the objectives, processes, and ethical issues discussed at these meetings as they relate to the WHO key ethical challenges. In addition, each of the co-authors of this paper attended at least one of the four meetings.

### Description of Processes Used in Organizing the Regional Meetings

To ensure that everyone had the basic knowledge needed to engage in the discussions, all meetings began with overviews of influenza biology, epidemiology, and history, including lessons learned from past influenza pandemics and the 2003 SARS pandemic. The focus of the meetings was on planning for a highly pathogenic H5N1 influenza; however, the H1N1 influenza was running its course during the Asian meeting and discussion of that epidemic entered the conversation. Information was presented on the 2007 WHO^18^ and 2007 CDC^14^ ethics guidance documents as a starting point for discussing how ethical considerations may differ between countries. In addition, there were presentations on the basic principles of public health ethics and how it differs from more traditional clinical and research ethics approaches, and discussion of ethical challenges that are likely to arise in response to planning for and responding to pandemic influenza. The Latin America meeting also included a session on human rights.

Out of respect for local partners, somewhat different processes were used in the four meetings to generate discussion of ethical issues. In the African meeting, participants met in small groups to discuss pre-developed case studies addressing the topics of non-pharmaceutical interventions, obligations of healthcare workers, and equitable access to treatment and prophylaxis. Participants were asked to consider the case in light of specific challenges they may face in their countries. In the Asian meeting, participants met in small groups and were asked to develop their own short narratives about the ethical issues they thought would be important in a pandemic influenza and how the values and cultural consideration in their counties would impact approaches for addressing these ethical issues. Participants wrote short narratives answering the following prompt:

*Prior to this meeting, a close friend explains that he does not understand why ethical issues are important in a pandemic response. What experience, either yours or someone else’s would you share to illustrate the significance and importance of public health ethics in a pandemic response?*

During the Latin America meeting, participants, grouped by country, were asked to identify key points for integrating ethics into emergency plans. During the Eastern Mediterranean meeting, participants were asked to share stories about ethical issues encountered during public health responses and to discuss how these issues were addressed in their response plans.

Organizers took minutes and notes of meeting proceedings and developed summary reports for all meetings. Unpublished reports are available at request.

## Results

For each of the organizing topics below, we first present some common themes discussed at the four meetings followed by more specific perspectives from each of the meetings in chronological order of when the meetings were held, beginning with African perspectives (August 2008), followed by Asian perspectives (March 2010), Latin American perspectives (July 2011), and Eastern Mediterranean (December 2011) perspectives. The WHO framework was used as a starting point for discussion at all meetings, but due to the characteristics, nature of events, and interests of local partners, discussions were not always focused on the same issues. Discussions at the two latter meetings were by-and-large affirming of the perspectives discussed at the two former meetings. Based on the meeting reports, fewer details related to the ethical challenges emerged from the Latin America and Eastern Mediterranean meetings than are for the Africa and Asian meetings.

### Transparency and Public Engagement

Transparency, in which relevant information is made freely available, and public engagement were seen by participants at all meetings as factors critical to an effective response during a pandemic influenza emergency. Many related issues were discussed, including low literacy level, poverty, and trust of and/or deference to health authorities. Some cultural variations were expressed; for example, that certain societies more readily accept autocratic directives for disease control. Participants at all meetings affirmed that their cultures do not tolerate corruption and indicated that a lack of transparency raises suspicions of corrupt dealings. Government authorities and leaders are expected to be open and consult the community in making important decisions, including public health emergency decisions, affecting their people. Factors that complicate mass communication that were discussed at all meetings include low levels of literacy, the inaccessibility of media such as television, newspapers, and the internet because of poverty; and the unavailability of the internet and cell phone towers in some rural areas. Although the detail and depth of discussions regionally varied and some cultural variation was evident, transparency in decision-making was in general decisively affirmed.

At the African meeting, in contrast to the general perception that “big men” and individuals with centralized power make all of the decisions, participants agreed that traditional cultures expect leaders to seek input from those they lead, through elder councils and similar institutions. Participants noted that public health leaders include traditional healers who serve as both recipients of and conduits for information. Because many Africans will seek care from traditional healers during a pandemic, these health providers must also be informed of how to protect themselves from infection, and how to guard against spreading the infection. In addition, a wide variety of local and international non-governmental organizations (NGOs), often funded by high-income countries, are active in resource poor countries in Africa seeking to meet the populations’ most basic needs. Thus, it is important that both traditional healers and NGOs be engaged in the decision-making process.

The participants at the Asian meeting varied widely in their views and practices relating to informing and engaging the public. For example, when SARS broke out in one Asian country, the government issued mandatory public health measures and expected public compliance. Due to the culture of deference to authority, nearly all communities in this country instantly adopted the measures (eg, quarantine, isolation, and social distancing). However, not all participants reported such deference to authority. This was reflected in a story from another Asian country about a boycott of a government polio immunization campaign by a minority community due to suspicions about the government’s motives. Others reported that when the central government was perceived as misgoverned or weak, responsibilities for informing the public about health threats and providing leadership during emergencies fell to local leaders.

Participants at the Latin American meeting stressed that community participation and cooperation will be crucial during a pandemic influenza response, particularly for migrants or minorities who are already stigmatized. Latin American participants also pointed to the importance of the media and health authorities in communicating health information and avoiding panic, as well as to convey factual information about availability and access to therapeutics. Some participants were concerned about the wide disparity in resources within and between countries, which make transparency even more important.

Participants at the Eastern Mediterranean meeting emphasized the need for inclusion, accountability, and transparency in public health policies, but also noted the reluctance among countries to collaborate because of political differences and disparity in wealth and resources. Participants discussed the need to establish a clear understanding of who will make what decisions during an emergency, how guidelines will be established, and the importance of considering multiple perspectives, including perspectives from individuals most at risk.

### Allocation of Scarce Resources

Economic, demographic, geographical, and population vulnerability factors were common challenges identified as affecting resource allocation decisions. These challenges were shared in all regions where the meetings occurred. Participants agreed that because of the cultural and regional variations, a “one size fits all” approach to any planning and response activity is unlikely to be optimal and should be questioned and challenged. However, although differences were acknowledged, there were also shared understanding and general agreement on the importance of the ethical values discussed at the meetings. Participants felt that planning and response should take into account contextual variations and cultural differences. Additionally, participants discussed the issue of resource allocation within the framework of transparency, especially when preferential treatment is given to the most powerful community members as opposed to the most vulnerable.

The African meeting participants affirmed the importance of providing resources to the young, but noted important differences in perspectives among countries. Many African societies give higher status to the elderly than to other age groups. With a life expectancy for some African countries in the 40s and even 30s, the proportion of the population composed of young children is much higher than in other countries. Thus, in African countries, a preference of allocating scarce resources to children would leave little resources for other segments of the population.

Generally, African countries are much more rural than are countries of other continents, making access to villages difficult, whether by road, telephone, or internet. Although meeting attendees did not feel that rural or urban habitation should be a criterion for allocation of resources, they anticipated that logistical challenges would make it so. These concerns are dwarfed, however, by the likelihood that resources such as antivirals and vaccine will be far scarcer in African countries than elsewhere because of the continent’s dual challenge of weak economies coupled with the number of other endemic health challenges, such as malaria and HIV/AIDS. With few resources at hand, the ethical imperative to respond to pandemic influenza may fall below that of addressing hyperendemic fatal diseases.

Each of the countries represented in the Asian meeting had a pandemic flu preparedness plan that addressed allocation issues and maintained a national stockpile of antiviral drugs. In most cases, biological vulnerability determined priority; thus, the very young or very old, pregnant women, and immune-compromised individuals tended to be prioritized to receive antiviral drugs. However, as Asian cultures exhibit more hierarchy than those in the West, more honor is accorded to the elderly, senior staff, royalty, and public service personnel in Asian cultures. In addition, it is expected that relatives and friends of the powerful will be unofficially prioritized to receive limited resources, without that being considered unethical. Indeed, in many Asian contexts, such prioritization is viewed as a social obligation (eg, a health worker would consider offering antivirals from the limited supply to a senior official before offering the resources to a person in one of the official priority groups). Nevertheless, participants expressed disapproval of officials who abuse their power and demand or extort limited resources for themselves. One participant described a shortage of N95 face masks (masks that can filter at least 95% of particles from the air) during the SARS epidemic. Although some people were willing to pay twice the regular price for a N95 mask, yielding to this demand would in effect favor protection for the rich over the poor. The participants felt the government had a duty to enforce price controls in order to ensure an adequate supply. As an example of enforcing price controls, one government instituted licensing for antiretroviral distribution to put a ceiling on the costs of the medications.

Some of the Asian perspectives on ethical distribution differed by religion. An appeal to Buddhist beliefs stated a priority for those who are most severely ill, and the young making sacrifices for their elders. A priority for women and children was expressed with reference to Catholic values. When a choice must be made between a mother and her child, participants at the Asian meeting felt that Catholic values would typically give preference to the mother.

Some Asian nations include island archipelagoes. It will be difficult for populations living on minor remote islands to access medical services and resources during a pandemic. The participants questioned whether their country plans address the challenges that certain geographical conditions may place on the equitable distribution of resources.

Many participants of the Latin American meeting thought that individual rights were paramount and that during a pandemic a clear communication plan that includes community input into the process for drug allocation would help avoid panic. They emphasized the importance of including all sectors of society, including the private sectors, migrants, and minorities in public health decision-making process. Issues such as discrimination and stigmatization of certain sectors of the population must be addressed before an emergency situation arises. Meeting participants stressed that emergency plans should take into account the diversity of population, must be transparent, and favor equal distribution of resources.

At the Eastern Mediterranean meeting, participants discussed the need to evaluate “what is good for you versus what is good for others.” This included discussing ethical challenges associated with distribution of scarce resources. Questions that were explored included: Which group of people should be vaccinated first? Who will make decisions about distribution? One theme that was identified from this discussion was the importance of prioritizing healthcare workers for access to limited resources, including medical and psychological care and other social benefits, should they become sick during an emergency.

### Social Distancing

The use of social distancing to limit transmission was widely accepted as an important tool in a pandemic influenza response, but participants warned of the many factors and challenges that complicate this traditional public health measure. These include socio-economic factors (eg, densely populated settings), and cultural factors (eg, family duty, funeral rituals).

Participants in the African meeting agreed with the social distancing principles described in the 2007 WHO document,^20^ including making the measures voluntary to the greatest extent possible; ensuring “safe, habitable, and humane conditions of confinement including the provision of basic necessities (food, water, clothing, medical care, etc)”; and employment protection for workers who comply with social distancing measures against the wishes of their employers. Participants stressed that isolation and quarantine measures will be more difficult to enforce in rural compared to urban areas in Africa due to the isolated geography of some rural areas and the lack of healthcare workers and security officials. However, participants noted that these public health measures have been successfully employed in rural areas in prior epidemics in Africa. Densely populated urban slums were also noted to present a challenge to social distancing. In a typical slum dwelling, people occupy all available sleeping space at night in small and poorly ventilated homes. There is no separate space available for isolation or quarantine. The same applies in some refugee camps. In such densely populated settings, the lack of freedom of movement may lead to near-certainty of transmission. Neighboring communities will be tempted to protect themselves by fencing off the slums or forcibly preventing the exit of slum residents. There was also concern among the participants that some African countries would rely heavily on military personnel to impose order, potentially with unnecessary force.

Participants in the Asian meeting also noted challenges associated with the use of social distancing measures. Duty to family is a major theme of Confucian philosophy. In some Asian countries, it is a tradition for friends and relatives to visit and even stay with a hospitalized person. In many instances, exhortations to family and friends about the serious nature of isolation are no match for the force of tradition: some find a way in and out of the isolation wards. Due to the lack of resources, isolation wards do not have security guards, and nursing staff are not able to add policing to their already heavy workload. A common concern reported by participants at the Asian meeting was the risk of stigmatization of patients and family members who were placed in isolation and quarantine. One of the participants reported that during the SARS outbreak, an entire village was stigmatized because it was home to one of the cases. Anybody known to have come from the village was avoided by others. Workers from the village were not admitted to their place of employment outside the village. Similarly, students were kept out of their schools. When the village was eventually quarantined, people feared delivering food and supplies. The stigma remained long after the epidemic subsided and the quarantine was lifted. High rates of poverty also pose a challenge for use of social distancing measures. It is difficult for patients to remain in isolation wards or for potentially exposed individuals to remain quarantined for long period of time unless compensation can be offered for lost wages.

Participants at the Latin American and Eastern Mediterranean meetings reflected on the long history of human rights abuses in their countries. This made them more likely to view use of social distancing measures as something that should be considered with great caution. Some even viewed these measures as human rights violations.

### Obligations to and of Healthcare Workers

Healthcare workers have multiple obligations, including obligations to their patients, to their employers, to their governments, to themselves, and to their families. Participants at all meetings understood that healthcare workers cannot completely sacrifice their and their family members’ health and well-being as they fulfill their public health duties during an emergency response.

Participants at the African meeting felt that healthcare workers have the right to stop working if they feel they are not well-protected. Factors discussed included the challenges related to the displacement of health workers during post-election conflict, traditional or cultural practices that may increase the risk of disease spread (eg, hugging or handshaking), and conflicts between senior officials and frontline healthcare workers regarding access to resources. They felt that frontline health workers should have first priority. A complicating factor in many African countries is the presence of large numbers of health-care-related NGOs from a variety of countries. What obligations would they have in a pandemic? If the workers or the organizations were to leave the country to attend to the needs of their home country or their families, the African country could lose a sizable proportion of its health workforce. And yet host governments have little authority to demand their assistance.

Socio-economic factors were predominant in stories told by participants at the Asian meeting. For example, during SARS outbreak, some private hospitals in one country were only admitting patients who could pay, while some suspected patients did not go to hospitals because they could not pay the inpatient care that could exceed $250 per day in a country where per capita annual income is less than $3000. Some countries reported lack of personal protective equipment, such as face masks, for healthcare workers; or differences in the degree of protection offered according to position (eg, physicians offered more protection than nurses). Participants reported that some healthcare workers refused to treat suspected cases, because either they did not have protective equipment and/or because they were concerned for their own safety and the safety of their loved ones to whom they would return after work. The participants agreed that healthcare facilities and governments had an ethical obligation to adequately and equitably provide personal protective equipment to their employees. In addition, participants felt that education of the employees about transmission control and, in some instances, additional incentives such as hazard pay, can help overcome the hesitancy of healthcare workers to remain on duty during a pandemic surge in cases.

Participants at the Latin American and Eastern Mediterranean meetings also discussed the roles of healthcare workers during influenza pandemic. Participants at both meetings recognized the important responsibilities healthcare workers have to treat patients regardless of the risk to themselves, but also noted that governments have responsibilities to protect healthcare workers. Some participants believed that healthcare workers have the right to refuse treatment to patients if the provider fears exposing their own family and that society has an obligation to compensate their families if they die while treating patients. Other participants felt that doctors do not have the right to refuse treatment because of their oaths and duties as physicians.

### International Collaboration

International collaboration is complicated by many factors, including disparities in resources, political differences, ethnic tension, and distrust. Participants, however, agreed that during a pandemic, collaboration is critical, because diseases respect no boundary. Participants pointed out that no country, developed or undeveloped, has eliminated poverty and the underlying causes of ill-health, such as lower literacy among the poor and less knowledge about disease prevention. The prevalence of poverty affects not only individuals, but institutions and systems. Because of the interdependence of nations, participants thought that it is in the best interest of resource rich countries to help build the capacity of poorer countries to conduct surveillance and disease control.

Participants in the African meeting stressed that the ability of a developing country to conduct thorough and accurate surveillance depends in large part on the assistance of developed countries in building and maintaining basic public health infrastructure well before a pandemic occurs. Moreover, by its very nature, surveillance is an ongoing process, not one that can be initiated in the face of an emergency response and then terminated when things return to normal. In emergencies, international scientists may temporarily fill some personnel gaps. Participants observed, however, that some international scientists providing technical assistance during an emergency seem more interested in research than in helping to control the disease outbreak. In some cases, they even diverted resources, such as healthcare workers, that could have been used for disease control. Lack of well-equipped laboratories in many African countries has resulted in the transfer of human biological specimens to distant laboratories, sometimes delaying diagnosis and intervention. Some surveillance resources are provided by donor nations for specific purposes such as measles eradication. Strict accounting rules may prevent the shift of those resources to other purposes, even in the face of a major global threat.

The Asian meeting participants also expressed concern about specimen sharing. During outbreaks of SARS and H5N1 influenza, for example, China shared its specimens with countries around the world for research and vaccine development.^33^ During the outbreak of H1N1 in 2009, WHO noted that 150 countries shared specimens.^34^ The Asian participants noted that collaboration and communication about disease transmission requires a transparency that can be at odds with the cultural value of ‘protecting honor’ and ‘avoiding being shamed’ that is common in Asia and elsewhere. Reporting an outbreak to other countries can be perceived as admitting inadequate disease control and asking for help from another country may be viewed as a sign of weakness. This is complicated by often pre-existing disputes between neighboring countries. Moreover, while helping others is also an important Asian cultural value, offering help when a country has not asked for it may be regarded as meddling with the internal affairs of that country. Moreover, two Asian countries who conducted a joint outbreak investigation exercise observed that multilateral coordination can be time-consuming in ways that hinder a speedy and effective response.

Participants at the Latin American meeting believed that it is important to clarify and disseminate guidelines for pandemic preparation and response, including those produced by WHO. Dissemination of pertinent information and guidelines between countries was considered as an obligation countries have to one another. Some noted that although wide disparity exists among Latin American countries, there is a great deal of solidarity, which facilitates cross-border collaboration, such as seen in the collaboration between Haiti and the Dominican Republic during the Haiti earthquake and resulting Cholera epidemic in 2010.

Participants at the Eastern Mediterranean meeting noted that countries are sometimes reluctant to collaborate because of the political and resource differences and other disparities between countries in the region. However, they agreed that plans for responding to an influenza pandemic should be shared among countries so that countries will be familiar with neighboring countries’ plans. Participants believed that country or even regional plans are too broad and more specific sub-regional plans should be developed and implemented.

## Discussion

Although the ethical concerns raised by participants from these four distinct regions (Africa, Asia, Latin American, and the Eastern Mediterranean) describe important issues that can shape responses to an international pandemic, the similarities of the perspectives and the concerns were notable. Participants reaffirmed the importance of the five key ethical issues framed by WHO (ie, transparency and public engagement, allocation of resources, social distancing, obligations to and of healthcare workers, and international collaboration). Participant feedback can be summarized as followed:

- The procedural values of transparency and inclusiveness are widely accepted and crucial for ethical decision-making.
- No “one size fits all” approach to allocating resources can address the variety of economic, cultural and other contextual factors that must be taken into account, but engaging with communities can help both to discover these factors and to build support for public health recommendations.
- Although meeting participants acknowledged the importance of social distancing as a tool to limit disease transmission, they also recognized the difficulties associated with this measure.
- Healthcare workers often have competing obligations that can compromise their ability to fulfill public health duties during an emergency response. Government has a responsibility to assist them in doing their job by providing appropriate training and equipment.
- Although international collaboration may be difficult, a focus on procedural ethics (ie, procedures that ensure transparency, consistency, inclusiveness, and a fair hearing of concerns in a deliberative format) make collaboration possible in efforts to combat global health threats.

The discussions from the meetings offer perspectives on how countries can collaborate in the control of international pandemics while respecting different cultural values. Although we initially were concerned that cultural differences could seriously impede international collaborations, we believed that anticipatory awareness of value differences would help prevent them from becoming potential stumbling blocks. Given this outlook, the meeting organizers were poised to highlight cultural differences. Indeed, the meeting exposed numerous cultural differences; eg, people in Asian countries more readily defer decision-making to government officials, elders, or other authority figures. Many of the differences that surfaced during the meetings reflected differences in how decisions are reached in the context of a country’s political arrangements. Moreover, as the discussion of resource allocation illustrated, differences in local contexts and traditions necessarily will play a role in how interventions will be implemented. Nevertheless, the similarities in perspectives between countries challenged our initial expectation that cultural differences would seriously impede if not prevent collaboration.

Attempts to change traditional cultural practices frequently fail or result in unintended consequences. However, addressing procedural ethics according to established international norms can assist with overcoming cultural differences within the context of global disease pandemic, political organization or local context. For example, the complicated ritual washing of bodies became a contentious issue in the 2014-2015 Ebola response, because of its role in facilitating the spread of Ebola virus. Culturally, this practice was considered an essential part of preparing the dead for the after-life.^35^ Public health and government workers contemplating halting or altering ritual practices require great cultural sensitivity and finesse in presenting alternatives that are perceived as fair and acceptable to a community already suffering from irreplaceable loss of their loved ones.

The recent WHO ethics workgroup on Ebola again illustrates the importance of a focus on procedural ethics.^36^ The workgroup included, along with ethicists and subject matter experts on Ebola, representatives from the three West African countries hit hardest by the Ebola virus. In relatively short order, the workgroup came to agreement on prominent ethical issues, such as the use of promising experimental drugs against Ebola virus, the need to conduct research on these drugs, and the importance of informed consent even during a public health emergency. Their success suggests that, when a fair process is established that includes the voices of those affected by the outbreak, a pandemic involving a deadly disease can bring countries rapidly together around the shared value of health, rather than divide them on the basis of cultural differences.

Perhaps the consensus regarding the importance of combating a pandemic health threat was to be expected, given that CDC or its partners such as Tephinet sponsored and coordinated all four meetings, and, more importantly, the 2007 WHO ethics guidance framed the discussions of ethics topics. Perhaps the participants consciously or subconsciously stated what they thought the sponsors wanted to hear. In addition, as many of the participants were public health officials, they brought with them a shared commitment to addressing health concerns. It is possible that the input of these health professionals, more numerous and vocal than other participants, explains the observed continuity around health-related matters. However, these conjectures do not seem compelling. CDC’s sponsorship and the framing of discussion around the WHO’s topics did not of themselves preclude major differences from surfacing within any particular topic. It also seems highly unlikely that the majority of differences were to be found outside of those ethics topics discussed at the meeting. The same WHO framework that oriented the participants to these topics also oriented them to the theme of cultural differences. Moreover, the exercises, discussions, and responses were open ended and varied rather than being highly directive. It also seems improbable that participants were merely telling us what we wanted to hear and held back from expressing profound differences when the purpose of the meetings was precisely to explore cultural differences.

A simpler and more compelling explanation lies in a cross-cultural continuity regarding the importance of combating the health threats that would result from a pandemic event. This continuity should come as no surprise. Human rights advocates, for example, deem health so fundamental to human flourishing that they consider it a basic human right.^37-41^ The “right of everyone to the enjoyment of the highest attainable standard of physical and mental health.” is encapsulated in article 12 of the *International Covenant on Economic, Social, and Cultural Rights* (CESCR), a covenant which 164 out of 197 countries have thus far ratified.^42,43^ Similarly, the capability approach maintains that the freedom to achieve well-being is a primary human capability that creates the opportunity for people to realize other capabilities they value. Campbell describes health as a liberation or freedom not only from pain or illness, but also as a freedom that allows a person to “create, inhabit a space, to simply live, and share the world around us.”^44^ For Campbell, the concept of health lies in this freedom. He further believes that the meaning of health reflects personal values and beliefs that are closely linked to the local community and socio-cultural group. Health in this view and as a matter of common human understanding is seen as a gateway, if not precondition, for developing other human functions and capabilities. It is a matter, then, not only of ethical theory but also of practical human life that pandemics, which pose existential threats to health, could be expected to elicit similar responses across cultures.

## Limitations

There are a number of limitations associated with this manuscript. The manuscript reflects what we found of most interest in the reports, notes, and stories generated from the four regional meetings. It does not provide a complete reporting of the meeting proceedings; rather, it focuses on the parts of the discussion that were related to the key ethical challenges identified in the WHO ethical framework document. The meetings were meant to initiate an international dialogue about how ethical considerations can be incorporated into pandemic influenza preparedness among members of the field epidemiology training programs, public health officials, policy-makers, scientists, ethicists, religious leaders, and representatives of international aid and health organizations. The meetings were not part of a research study meant to develop new or generalizable knowledge. Participants were not recruited in a systematic fashion. CDC relied on local partners to identify and nominate participants to attend the regional meetings. Meeting agendas, sessions, and structures were tailored to the local interests and circumstances. Although participants were oriented to the WHO ethics framework at the beginning of each meeting, discussions were not always focused on the same issues. This may explain the lack of consistency in the amount and depth of the discussions on the key ethical challenges identified in the WHO document.

## Conclusion

If the analysis and explanations above are sound, then it indicates that cultural differences need not pose a serious challenge for collaboration between countries in addressing an international pandemic. Likewise, substantive ethical differences need not pose a serious impediment to pandemic preparedness efforts especially if more attention is paid to procedural ethics, that is, to procedures that ensure transparency, consistency, inclusiveness, and a fair hearing of concerns in a deliberative format.^45^

If any lesson is learned from past pandemics, it is that each one informs our response to the next. Likewise, the ethical issues raised by past public health emergencies should serve to better prepare ourselves to effectively respond to the next emergency.^46^ The same applies to the discussions generated by the regional meetings described in this document. They affirm the notion that, cultural differences notwithstanding, people and countries will come together to combat the health threat a pandemic influenza poses to all, when fair procedures are established that give those affected a seat at the table and a voice.

## Acknowledgements

We acknowledge the invaluable contributions of the following individuals and organizations in organizing the regional meetings, developing meeting reports and summaries, from which this manuscript was based. We especially appreciate the work of Mark White (CDC retired) who conceptualized, designed, collected, and analyzed data, and obtained funding and coordinated the regional meetings with external partners. In no particular order, we also would like to acknowledge the contributions of Maria Consorcia Lim- Quizon, David Mukanga, Fred Wabwire-Mangen, Joseph Ochieng, Patrick Nguku, Rebecca Babirye, Dominic Thomas, Anant Bhan, Goldie MacDonald, Andreas Reis (WHO), AFENET, EMPHNET, SAFETYNET, and TEPHINET.

## Disclaimer

The findings and conclusions in this paper are those of the authors and do not necessarily represent the official position of the Centers for Disease Control and Prevention (CDC), the University of North Carolina, Chapel Hill, NC, USA or TEPHINET at the Task Force for Global Health.

## Authors’ contributions

All coauthors were involved in reviewing reports, stories, summaries, and notes from the meetings described in the manuscript.

## Competing interests

Funding was provided by the United States Agency for International Development and CDC to support the four regional meetings described in the manuscript. Funding was used for travel-related expenses of all participants, including the co-authors of this paper, to participate in one or more of the meetings.

## Ethical issues

Not applicable. This manuscript was based on analysis of reports and documentations generated from four CDC-sponsored regional meetings. The meetings were considered routine public health practice and not research per se.

## Authors’ affiliations

^1^Center for Global Health, Centers for Disease Control and Prevention, Atlanta, GA, USA. ^2^Gilllings School of Global Public Health, University of North Carolina, Chapel Hill, NC, USA. ^3^Office of Science Integrity, Office of the Associate Director for Science, Centers for Disease Control and Prevention, Atlanta, GA, USA. ^4^Training Programs in Epidemiology and Public Health Interventions Network, Task Force for Global Health Inc., Atlanta, GA, USA.

## Key messages

**Implications for policy makers**

- The procedural values of transparency and inclusiveness are widely accepted and crucial for ethical decision-making.
- No “one size fits all” approach to allocating resources can cover the variety of economic, cultural and other contextual factors that must be taken into account, but engaging with communities can help both to discover these factors and to get buy in.
- Although meeting participants acknowledged the importance of social distancing as a tool to limit disease transmission, they also recognized the difficulties associated with this measure.
- Healthcare workers often have competing obligations that can compromise their ability to fulfill public health duties during an emergency response. Government has a responsibility to assist them in doing their job by providing appropriate training and equipment.
- Although international collaboration can be difficult, focusing on shared values and fair procedures (procedural ethics) can bring countries together to combat the common health threat that global pandemics pose.

**Implications for public**

Recognizing that cultural perspectives may shape the ethical context of an emergency response, the Centers for Disease Control and Prevention (CDC) funded four regional meetings to explore cultural perspectives relevant to pandemic influenza preparedness and response. Although regional cultural differences were observed, these differences will not prevent countries from coming together to collectively address a shared existential health threat. Pandemics create a global predicament that can unite countries around the shared value of health, rather than divide on the basis of cultural differences.
